# Intertemporal Choice Behavior in Emerging Adults and Adults: Effects of Age Interact with Alcohol Use and Family History Status

**DOI:** 10.3389/fnhum.2015.00627

**Published:** 2015-11-23

**Authors:** Christopher T. Smith, Eleanor A. Steel, Michael H. Parrish, Mary K. Kelm, Charlotte A. Boettiger

**Affiliations:** ^1^Neurobiology Curriculum, University of North Carolina, Chapel HillNC, USA; ^2^Department of Psychology and Neuroscience, University of North Carolina, Chapel HillNC, USA; ^3^Bowles Center for Alcohol Studies, University of North Carolina, Chapel HillNC, USA; ^4^Biomedical Research Imaging Center, University of North Carolina, Chapel HillNC, USA

**Keywords:** alcoholism, family history, decision-making, delay-discounting, impulsivity

## Abstract

Adults with alcohol use disorders (AUDs) show marked immediate reward selection (or “*Now*”) bias in intertemporal choice tasks. This *Now* bias persists long into abstinence, suggesting an irreversible consequence of chronic alcohol abuse or a pre-existing AUD intermediate phenotype. However, some data show substantial *Now* bias among emerging adults (18–25), regardless of drinking behavior, suggesting age-dependent effects on *Now* bias. The objectives of the present study were to determine (1) whether *Now* bias is greater among emerging adults relative to adults, (2) whether any such age effect on *Now* bias is diminished in sub-clinical heavy alcohol users, and (3) whether having a problem drinking first degree relative is independently associated with elevated *Now* bias. To achieve these objectives, we used an intertemporal choice task to quantify *Now* bias in *n* = 237 healthy participants (ages 18–40; 50% female), and a wide range of non-zero alcohol use, based on the Alcohol Use Disorders Identification Test (AUDIT). We found that among non-heavy drinkers, *Now* bias inversely correlated with age; this relationship was not present among heavy drinkers. We found no significant relationship between AUDIT score and *Now* bias among emerging adults, but AUDIT scores and *Now* bias were positively correlated among 26–40 year olds. Additionally, non-heavy drinking adults who reported a problem drinking first degree relative showed greater *Now* bias compared to those not reporting familial problem drinking. While not definitive, these findings lend support for elevated *Now* bias in adulthood as an intermediate phenotype for AUDs. Moreover, non-additive effects of age and heavy drinking on *Now* bias suggest perturbations in largely common neural circuits in both groups.

## Introduction

All individuals tend to discount the value of delayed reward to some degree (Mazur, 1987; Rachlin, 2000), however, adults with addictive disorders, including alcohol use disorders (AUDs), tend to choose smaller, sooner over larger, delayed reward in the context of intertemporal choice (or “delay-discounting") tasks more frequently than do adults with no addiction history (Vuchinich and Simpson, 1998; Petry, 2001; Mitchell et al., 2005; MacKillop et al., 2011). This immediate reward selection (or “*Now*”) bias persists even after years of abstinence and does not correlate with abstinence duration (Mitchell et al., 2005), suggesting irreversible consequences of chronic alcohol abuse and/or a pre-existing risk trait, or intermediate phenotype (Meyer-Lindenberg and Weinberger, 2006; MacKillop, 2013; Bickel, 2015). This possibility is further supported by data showing that people with other conditions characterized by impulsive behavior also exhibit elevated *Now* bias, including attention deficit hyperactivity disorder (Barkley et al., 2001; Sonuga-Barke et al., 2008; Paloyelis et al., 2010), and pathological gambling (Alessi and Petry, 2003; Dixon et al., 2003; MacKillop et al., 2011; Leeman and Potenza, 2012). If heightened *Now* bias is an AUD intermediate phenotype, we would predict heightened *Now* bias among people who engage in heavy, at-risk drinking but who do not meet clinical criteria for alcohol dependence, relative to age-matched non-heavy drinkers. We would also predict heightened *Now* bias among non-heavy drinkers with problem-drinking first degree relatives.

Data indicating that *Now* bias is highly heritable (Anokhin et al., 2011; Mitchell, 2011) lends further support to the idea of *Now* bias an intermediate phenotype for AUDs. However, intertemporal choice is known to involve frontal structures (Boettiger et al., 2007, 2009; Bjork et al., 2009; Kim and Lee, 2011; Hare et al., 2014; Wesley and Bickel, 2014; Behan et al., 2015; Cho et al., 2015; Massar et al., 2015), and the development of frontal structures remains incomplete until the early-to-mid twenties (Sowell et al., 1999; Casey et al., 2000; Sowell et al., 2001; Giedd, 2004; Gogtay et al., 2004; Lenroot and Giedd, 2006). As such, developmental processes may potentially occlude the detection of elevated *Now* bias in younger individuals at greater risk for AUDs. Indeed, we have previously found marked *Now* bias among emerging adults (18–25 years), regardless of drinking behavior (Kelm et al., 2010). This suggests elevated *Now* bias generally among individuals transitioning from adolescence to adulthood. The prior observation that healthy adults with no AUD diagnosis display reduced *Now* bias compared to abstinent alcoholic adults (Mitchell et al., 2005; Boettiger et al., 2007) suggests that *Now* bias should decline as a function of age between emerging adulthood and adulthood only among moderate drinkers. While emerging adults are widely regarded as impulsive (Chambers and Potenza, 2003; de Wit, 2009), and *Now* bias is normally higher in childhood than in the early 30’s (Green et al., 1994; Scheres et al., 2006; Olson et al., 2007; Eppinger et al., 2012), little is known about *Now* bias changes specific to the period from emerging adulthood to adulthood. A structural neuroimaging study of individuals aged 9–23 found that age-related increases in frontal white matter integrity negatively correlate with *Now* bias (Olson et al., 2009), and while white matter changes from late adolescence to early adulthood were not examined, these data demonstrate that maturation of frontal structures occurs concurrently with age-related declines in *Now* bias. Thus, it is possible that continued maturation of frontal circuits contributes to continued decline in *Now* bias from emerging to full adulthood. *Now* bias positively correlates with trait impulsivity measures (Mitchell et al., 2005; de Wit et al., 2007), which decline linearly with age from early adolescence to age 30 (Steinberg et al., 2008). Thus, we expect less *Now* bias among adults relative to emerging adults, but, to our knowledge, prior studies have not explicitly investigated age effects on *Now* bias from ages 18 to 40. Moreover, whether any such age-related changes in *Now* bias interact with heavy alcohol use is unknown.

We tested whether an age-related decrease in *Now* bias occurs from emerging adulthood to adulthood, and whether any such age effect is absent among sub-clinical heavy drinkers. To do so, we recruited individuals aged 18–40 with no substance use disorder (SUD) history who had consumed alcohol at least once. Approximately half were early emerging adults (18–21) and half were adults (22–40). We recruited these age groups based on a prior finding that a genetic regulator of *Now* bias differentially affects the intertemporal choice behavior of these groups (Smith and Boettiger, 2012), and data showing that brain maturation asymptotes at ∼22 years (Dosenbach et al., 2010). We used Alcohol Use Disorders Identification Test (AUDIT; Saunders et al., 1993) scores to recruit equal numbers of light/moderate drinkers (AUDIT scores <8 for males and <5 for females; Neumann et al., 2004) and heavy drinkers (AUDIT scores ≥8 for males and ≥5 for females) within each age group. Advantages of using the AUDIT include its demonstrated validity across cultures, age and genders (Saunders et al., 1993; Allen et al., 1997; Cherpitel, 1998), coupled with its brevity and simplicity. While full AUDIT scale scores were used for recruitment, the AUDIT includes three subscales, alcohol consumption, alcohol dependence, and alcohol-related harm, which we also used in some analyses. We quantified *Now* bias using a previously validated intertemporal choice task (Mitchell et al., 2005; Altamirano et al., 2011) optimized for neuroimaging and known to engage the frontal lobes (Boettiger et al., 2007; Kayser et al., 2012) and assessed the effects of age, alcohol use, their interaction, and family history (FH) of alcoholism on *Now* bias. Examining these relationships may offer additional insight into the utility of *Now* bias as an intermediate phenotype for AUDs.

## Materials and Methods

### Participants

The University of North Carolina (UNC) Office for Human Research Ethics approved this study. Subjects provided written, informed consent. Participants (*n* = 246; ∼50% female) aged 18–40 were recruited from UNC and surrounding community. We recruited participants based on AUDIT scores (Saunders et al., 1993), age, and sex obtained via a phone-based screening session. The Heavy Drinker groups were defined by AUDIT scores ≥8 for males, and ≥5 for females (*n* = 142, mean: 11.8 ± 4.7), and the Non-heavy Drinker groups had AUDIT scores <8 for males and <5 for females (*n* = 104, mean: 3.3 ± 2.1; Neumann et al., 2004). The “early emerging adult” group included participants ages 18–21 and our “adult” recruitment group included participants ages 22–40. Participants self-reported consuming alcohol one or more times in their lifetime, had no known history of any neurological, SUD, or other psychiatric disorders, and no current psychoactive drug use, excluding nicotine, caffeine, and alcohol. Behavioral inventories, intertemporal choice task, and saliva samples for genetic analysis were obtained during a single in-person experimental session. Although no participants self-reported any AUD, *post hoc* evaluation of responses in the Rutgers Alcohol Problem Index (RAPI) indicated probable alcohol dependence among 43 recruited subjects (17.5%; 91% Heavy drinkers), based on *Diagnostic and Statistical Manual of Mental Disorders* (fourth ed.; *DSM–IV*) criteria (American Psychiatric Association, 1994); however, excluding these participants from our analyses did not qualitatively change our findings. Therefore, we did not exclude these participants, except where explicitly noted. Nine subjects were excluded from all analyses due to unreliable task performance (see below). Thus, 237 participants (*n* = 118 male) are included in our analyses; sex ratios were balanced within each recruitment group. Behavioral Inventories.

We administered standard questionnaires to quantify personal substance use, alcoholism familial history (FH), and behavioral traits. These included the AUDIT, the RAPI (White and Labouvie, 1989), the Drug Abuse Screening Test (DAST) (Skinner, 1982), and the Drug Use Screening Inventory, Domain I (DUSI-I); (Tarter, 1990). DUSI-I scores reported as % affirmative answers from Domain I, part B. We calculated density of familial alcohol abuse from the Family Tree Questionnaire (FTQ) (Mann et al., 1985), and classified participants reporting a problem drinking father or sibling as FH positive for alcoholism (FHP; *n* = 76). Those reporting a problem-drinking mother (*n* = 22) were excluded from our FH analyses to avoid potential confounds from possible fetal alcohol exposure. Those reporting no problem drinking first degree relatives were classified as family history negative (FHN; *n* = 161). The Barratt Impulsiveness Scale-11 (BIS); (Patton et al., 1995) was used as a subjective measure of trait impulsiveness. Socio-economic Status (SES) was quantified as Hollingshead scores, following the Barratt Simplified Measure of Socioeconomic Status method (Hollingshead, 1975; Barratt, 2006).

### Intertemporal Choice Task

The task has been described in detail previously (Altamirano et al., 2011; Smith and Boettiger, 2012). In brief, subjects practiced, then completed eight blocks of 42 trials. There were four conditions: WANT, DON’T WANT, SOONER, and LARGER; the latter two are considered together as control (CON) trials. Trial types were pseudorandomly ordered. Each trial displayed two monetary reward options, one ($2–$100) available at a delay (1 week to 6 months) and a lesser amount (5–30% less) available “TODAY.” All choices were hypothetical. Participants chose their preferred option on WANT trials, their non-preferred option on the DON’T WANT trials, and the side with the sooner time or larger monetary amount for SOONER and LARGER trials, respectively. The delayed amount, delay time, percent discount, and left/right position were pseudorandomly selected for each trial. We also collected reaction time (RT) for each trial. Nine subjects were excluded based on faster RT in the WANT and/or DON’T W trials than in the CON trials, indicating lack of subjective consideration of options.

### Genotyping

We previously found that a polymorphism in the catechol-*O*-methyltransferase (COMT) gene (*COMTval^158^met; rs4680)* interacts with age to affect *Now* bias (Smith and Boettiger, 2012). To control for this potential confound, participants were genotyped for the *COMTval^158^met* polymorphism as previously described (Smith and Boettiger, 2012; Kelm and Boettiger, 2013; Smith et al., 2014; Swift-Scanlan et al., 2014). Although COMT genotype distribution did not differ across recruitment groups (see **Supplementary Tables S1**–**S3**), we included a COMT^∗^age covariate in our analyses to account for the COMT by age effect we previously observed.

### Data Analysis

Our primary index of *Now* bias is the proportion of smaller, sooner choices made in the W condition, the impulsive choice ratio (ICR). Inferred ICR (*i*ICR) at each delay time was calculated based on the option that was not selected in DON’T WANT trials. We calculated the absolute difference between ICR and *i*ICR at each delay time, and averaged this value across all delay times as a gross index of motor control (motor mismatch, MM; Mitchell et al., 2007).

For single factor statistical comparisons between groups, we used unpaired two-tailed *t*-tests for continuous measures and χ^2^ tests for categorical measures. For multi-factorial comparisons, we used mixed model ANOVA using SPSS (IBM, Montauk, NY, USA). When necessary, a Greenhouse–Geisser non-sphericity correction was applied. When data were not normally distributed, arcsine-root transformations were applied in Excel prior to statistical tests to ensure the validity of parametric statistical tests. All analyses performed in SPSS unless otherwise noted. Effect sizes for ANOVA are reported as η^2^, while effect sizes for *t*-tests are reported as Cohen’s *d.*

### *Post Hoc* Analyses to Maximize Distinction between Age and Alcohol-use Groups

Our recruitment groups were empirically based, but the age cut-offs between groups may not be optimal for detecting age effects on ICR. To inform future studies requiring smaller sample sizes, we used signal detection theory to identify, *post hoc*, age cutoff scores producing the largest group difference in ICR. To identify the age cutoff that maximized our ability to discriminate emerging adults from “full” adults in terms of mean ICR, we compared the magnitude of our discriminability index, *d*′ (Green and Swets, 1966), across different age cutoffs in low AUDIT individuals, as this was the sample in which we initially observed significant effects of age on ICR. We evaluated age cutoffs in the Low AUDIT recruitment sample by grouping participants into older and younger age groups with a gap year between groups. We then used a sliding window to calculate ICR means and SDs for each age group pair for each age cutoff (from ages 21–31). We calculated *d*′ for ICR group differences as:

$$d^{\prime }=\frac{\left(2\times ({ICR}_{group1}-{ICR}_{group2})\right)}{\sqrt{{\left({SD}_{ICRgroup1}\right)}^{2}+{\left({SD}_{ICRgroup2}\right)}^{2}}}$$

where ICR_group_*_n_* and SD_ICRgroup_*_n_* are the ICR mean and standard deviation for group *n*, respectively. We confirmed the *d′* findings with Cohen’s *d* effect sizes calculations (Cohen, 1988) using a pooled measure of SD (Hartung et al., 2008). Maximal discrimination of ICR between age groups occurred with an adult age cutoff of 26 (i.e., comparing ages 18–24 to ages 26–40; **Supplementary Figure S1**). Cohen’s *d* age group effect sizes were also largest with an age cutoff of 26 (Cohen’s *d* = 0.82). Based on these discriminability results, our age group analyses were based on classification of participants as emerging adults (ages 18–24, *n* = 184; mean age = 20.8 ± 1.7) or adults (ages 26–40, *n* = 39; mean age = 31.4 ± 4.0). We used the same equation and a similar approach to identify optimal AUDIT-c cut-off values in our adult group.

## Results

### Demographic and Psychometric Data

Based on preliminary data from our lab, we initially recruited early emerging adult (ages 18–21) and young adult (ages 22–40) subjects, with roughly equal ratios of at-risk drinkers within each group. Recruited age groups did not differ in terms of ethnicity, sex, SES, FH of alcohol abuse, measures of substance use, or *COMT* genotype distribution (**Supplementary Table S1**), nor did AUDIT recruitment groups (**Supplementary Table S2**). Finally, we detected no significant interaction between age and AUDIT recruitment groups in terms of demographics, but did find greater intensity of substance use among 18–21 year olds relative to 22–40 year olds within the high AUDIT group (**Supplementary Table S3**).

We investigated whether any demographic, substance use, or psychometric measures varied across our emerging adult (18–24), adult (26–40) and low and high AUDIT recruitment groups via a 2 × 2 ANOVA. We found that these four groups did not differ in terms of ethnicity, sex, SES, FH of alcohol abuse, or *COMT* genotype distribution (**Table 1**). Moreover, we found no significant age*AUDIT group interactions on any measure except AUDIT and DUSI scores (**Table 1**). This result reflects ∼47% higher AUDIT scores and ∼39% higher DUSI scores among 18–24 year olds relative to adults within the high AUDIT group (**Table 1**).

**Table 1:** Demographic, substance use related, and psychometrics measures across age and drinking groups.

	Non-heavy drinkers		Heavy drinkers		Age^∗^drinking interaction	
			
	Ages 18–24 (*n* = 75)	Ages 26–40 (*n* = 20)	Ages 18–24 (*n* = 109)	Ages 26–40 (*n* = 19)	*F*_219_	*p*
**Demographic**						
Age (years)	20.7 ± 1.7	31.2 ± 4.0	20.9 ± 1.7	31.5 ± 4.2	0.02	0.89
Education (years)	14.7 ± 1.6	17.6 ± 1.5	14.7 ± 1.5	17.6 ± 2.8	<0.001^a^	0.998
SES	50.6 ± 10	53.1 ± 7.7	51.4 ± 7.7	52.9 ± 9.6	0.13	0.71
Ethnicity (% non-white)	25.3	40.0	29.4	26.3		0.63^†^
Sex (% female)	42.7	55.0	52.3	63.2		0.34^†^
COMT genotype (% ValVal)	22.7	30.0	29.4	36.8		0.73^†^
**Substance use-related**						
AUDIT – total	3.5 ± 2.2	3.0 ± 1.9	12.5 ± 4.8	8.5 ± 2.5	7.19	0.008
AUDIT consumption	3.0 ± 1.4	2.7 ± 1.4	6.6 ± 1.9	4.8 ± 1.1	6.53^b^	0.011
AUDIT dependence/harm	0.9 ± 1.2	0.6 ± 1.2	6.2 ± 3.8	3.7 ± 2.3	4.77^b^	0.03
RAPI	2.9 ± 4.0	1.5 ± 3.7	12.3 ± 7.5	7.9 ± 7.7	1.77	0.19
DUSI	0.12 ± 0.13	0.12 ± 0.13	0.39 ± 0.15	0.28 ± 0.19	4.44	0.04
DAST	1.0 ± 1.4	0.85 ± 0.9	2.7 ± 2.7	2.6 ± 3.5	<0.001	0.99
FTQ density (%)	13.9 ± 15.5	21.5 ± 19.5	15.4 ± 16.8	22.2 ± 20.8	0.019	0.89
**Psychometric**						
BIS – total	56.9 ± 8.7	55.7 ± 9.8	61.7 ± 9.4	55.3 ± 9.9	2.55^c^	0.11
BIS Attention	15.4 ± 3.0	14.4 ± 4.0	16.1 ± 3.7	14.6 ± 3.8	0.16^c^	0.69
BIS Motor	21.2 ± 3.5	20.8 ± 3.1	22.4 ± 3.8	20.0 ± 3.3	2.45^c^	0.12
BIS non-planning	20.3 ± 4.3	20.6 ± 5.0	23.2 ± 4.5	20.7 ± 4.6	2.95^c^	0.09
FTPI mean ext (years)	8.6 ± 5.3	5.1 ± 4.0	7.5 ± 5.8	6.9 ± 5.0	2.24	0.14
FTPI max ext (years)	30.7 ± 21.8	19.8 ± 15.2	28.0 ± 23.6	23.1 ± 14.2	0.62	0.43


*Values are reported as mean ± standard deviation. Reported *F* and p-values reflect the results testing for significant age by AUDIT group interactions. Exact p-values reported unless *p* < 0.001. ^†^*p*-value represents results of χ^2^ test. ^a^error = 217, ^b^error = 202; ^c^error = 218.*

### AUDIT Does Not Predict *Now* Bias (ICR) in Emerging Adults

In contrast to findings of a direct relationship between ICR and alcohol use in adult samples (Mitchell et al., 2005, 2007; Boettiger et al., 2007), among emerging adults (ages 18–24), we found no significant difference in ICR between high AUDIT (0.63 ± 0.32) and low AUDIT (0.68 ± 0.25) groups [*t*_(180.448)_ = 1.29, *p* = 0.20, *d* = 0.18]. Moreover, in considering AUDIT as a continuous variable, among emerging adults, we observed no significant correlation between ICR and AUDIT [*r*_(182)_ = 0.014, *p* = 0.42, β = 0.001] or with any other substance use measure (maximum *r* = 0.065, min. *p* = 0.383).

### Interacting Effect of Age and Heavy Drinking on *Now* Bias

**FIGURE 1:**
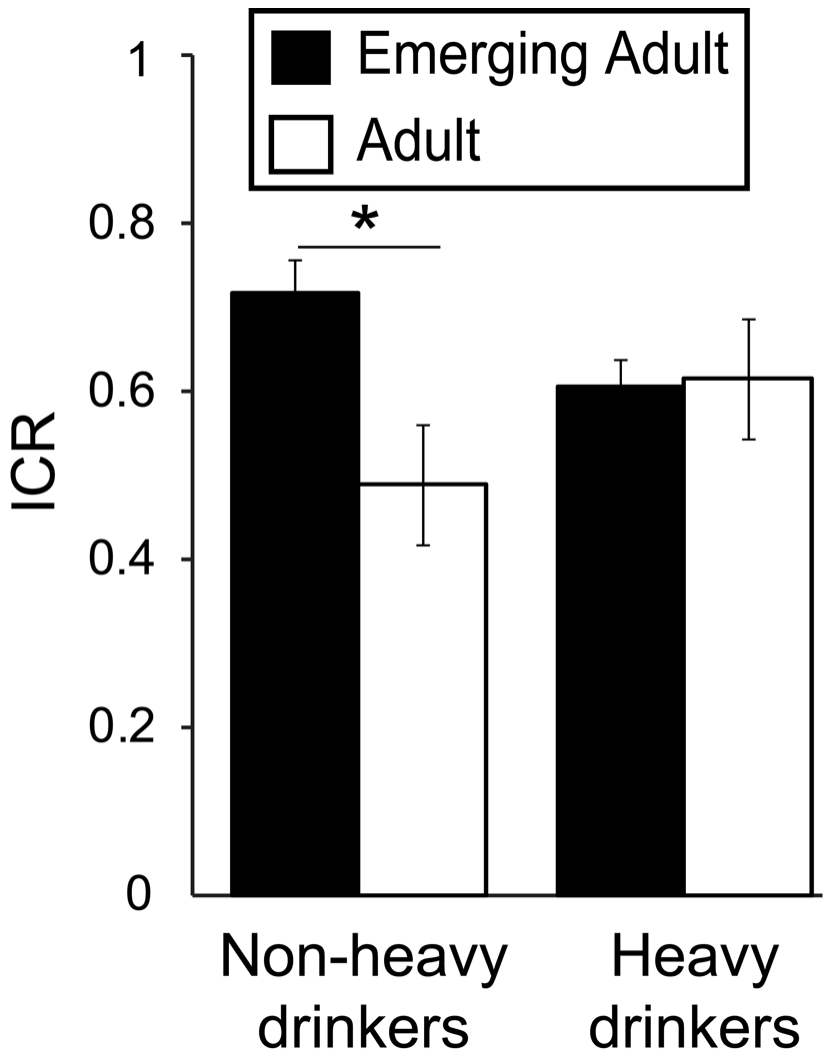
**Interacting effects of age and alcohol use on *Now* bias.** Plot depicts the ratio of immediate reward choices (ICR) in the delay-discounting task as a function of drinking (heavy/non-heavy) and age (adult/emerging adult) groups, demonstrating a significant age^∗^drinking interaction [*F*_(1,217)_ = 5.17, *p* = 0.024, η^2^ = 0.023]. This finding reflects the fact that among non-heavy drinkers, ICR was significantly higher in emerging adults relative to adults [*F*_(1,91)_ = 8.46, *p* = 0.005, η^2^ = 0.085], but among heavy drinkers, ICR did not differ between age groups. AUDIT, Alcohol Use Disorders Identification Test. ^∗^*p* < 0.05.

Based on our hypothesized age-related decrease in ICR specific to non-heavy drinkers, we conducted a two-way (age group*drinking group) ANOVA, covarying for drug use (DUSI scores). We did not detect a significant main effect of heavy drinking [*F*_(1,217)_ = 0.042, *p* = 0.84, η^2^ < 0.001], but did find a trend toward a main effect of age group [*F*_(1,217)_ = 3.66, *p* = 0.057, η^2^ = 0.016] and a significant age^∗^heavy drinking interaction [*F*_(1,217)_ = 5.17, *p* = 0.024, η^2^ = 0.023; **Figure 1**). *Post hoc* analyses found that among non-heavy drinkers, mean ICR was ∼48% higher in 18–24 year olds (0.68 ± 0.25) relative to 26–40 year olds [0.46 ± 0.37; *F*_(1,91)_ = 8.46, *p* = 0.005, η^2^ = 0.085], which survives Bonferroni correction for multiple comparisons (*p* < 0.025). In contrast, in heavy drinkers, mean ICR was less than 2% higher in 18–24 year olds (0.63 ± 0.32) relative to those ages 26–40 [0.62 ± 0.32; *F_(_*_1,124)_ = 0.009, *p* = 0.93, η^2^* <* 0.001].

### Among Non-heavy Drinkers, *Now* Bias Varies Inversely with Age

While we observed a significant effect of age group on *Now* bias among non-heavy drinkers, we also performed an alternative, and more statistically powerful correlation analysis, treating age as a continuous variable, rather than as a dichotomous group variable. In the full sample, we found a significant negative correlation between ICR and age [*r_(_*_235)_ = -0.134, *p* = 0.019, β = -0.012) indicating ∼1.2% lower ICR for each additional year of age. This age effect was driven by non-heavy drinkers, who showed ∼2.2% lower ICR with each additional year of age [*r*_(97)_ = -0.276, *p* = 0.003, β = -0.022], which survives Bonferroni correction for multiple comparisons (*p* < 0.025). In contrast, we observed no significant relationship between ICR and age among heavy drinkers [*r*_(136)_ = -0.025, *p* = 0.39, β = -0.002]. Thus, parsing our data either according to *a priori* age groups or considering age as a continuous variable yields the same finding: ICR inversely relates to age in non-heavy drinkers, but no consistent relationship between ICR and age is observed in heavy drinkers.

### Other Aspects of Behavioral Task Performance

Importantly, we found no significant main or interacting effects of age or drinking group on basic measures of task performance, including accuracy in control trials, and RTs across conditions (**Table 2**). Moreover, we found no significant main or interacting effects of age or drinking group on unintentional motor responding (MM; see Materials and Methods; maximum *F* = 0.785, minimum *p* = 0.38). Thus, group differences in motor impulsiveness cannot explain group differences in *Now* bias observed here. A recent study reported greater *Now* bias in adolescents (ages 13–15) relative to adults (ages 19–50) that was associated with greater choice inconsistency (Ripke et al., 2012). Here, we found that the consistency of ICR across task blocks (Eight blocks total; 42 trials per block) did not differ between age groups (Cronbach’s *α*, emerging adult: 0.98, adult: 0.99). Furthermore, a two-way repeated measures ANOVA (age group^∗^block) found no significant main effect of block nor block^∗^age group interaction (maximum *F* < 1, minimum *p* > 0.46) on ICR. Thus, the age effects on *Now* bias reported here are not attributable to age–related changes in response consistency. Task related performance and these other measures of discounting are reported across drinking and age groups in **Table 2**. We saw no significant age^∗^drinking group interaction on any performance related measure and the only significant interaction was observed with our AUC measure, an alternate *Now* bias metric.

**Table 2:** Other delay discounting task measures across age and drinking groups.

	Non-heavy drinkers		Heavy drinkers		Age^∗^drinking interaction	
			
	Ages 18–24 (*n* = 75)	Ages 26–40 (*n* = 20)	Ages 18–24 (*n* = 109)	Ages 26–40 (*n* = 19)	*F*_219_	*p*
**Task performance**						
Control trial Acc	97.0 ± 3.3	96.7 ± 3.5	96.6 ± 3.6	98.1 ± 2.0	2.22	0.14
Control trial RT	1364 ± 339	1347 ± 315	1401 ± 329	1233 ± 276	1.68	0.20
WANT trial RT	1920 ± 383	1743 ± 388	1915 ± 396	1711 ± 389	0.04	0.84
DON’T WANT trial RT	2064 ± 399	1859 ± 385	2086 ± 449	1888 ± 369	0.002	0.96
**Other task measures**						
Motor mismatch	0.11 ± 0.06	0.10 ± 0.07	0.11 ± 0.06	0.12 ± 0.07	0.61	0.44
ICR consistency (α)	0.978	0.991	0.987	0.988	0.86^†^	0.51^†^


*Values are reported as mean ± standard deviation. Reported *F* and p-values reflect the results testing for significant age by AUDIT group interactions. Exact p-values reported unless *p* < 0.001. Conventions as per **Table 1**. ^a^error = 210. ^†^result of ICR by Block by Age by AUDIT Group ANOVA, Greenhouse–Geisser corrected: *F*_*(7,1477)*_.*

### Relationship between AUDIT Scores and *Now* Bias: Moderation by Age

We further investigated the apparent lack of age effect on *Now* bias among heavy drinkers by evaluating the relationship between ICR and AUDIT scores within age groups. In contrast to 18–24 year olds, among 26–40 year olds, we found a positive correlation between AUDIT score and ICR (**Table 3**). This relationship appears to be primarily driven by a relationship between ICR and AUDIT consumption subscale (AUDIT-c) scores, as we observed no significant relationship between ICR and AUDIT dependence/harm subscales in adults (**Table 3**). Among 18–24 year olds, we found no significant relationship between ICR and AUDIT subscale scores (**Table 3**). In adults 26-40, each additional AUDIT-c point was associated with a 15.6% increase (β = 0.117) in ICR (**Table 3**). In contrast, increases in AUDIT-c scores had no significant effect on ICR among emerging adults (18–24; **Table 3**).

**Table 3:** Alcohol Use Disorders Identification Test correlates with ICR more strongly in adults and is driven by AUDIT consumption subscale.

	AUDIT total	AUDIT consumption	AUDIT dependence/harm
18–24	*r*_(184)_ = 0.014	*r*_(166)_ = 0.018	*r*_(166)_ = 0.017
(*n* = 184)	*p* = 0.42	*p* = 0.41	*p* = 0.41
	β = 0.001	β = 0.003	β = 0.002
26–40	*r*_(37)_ = 0.30	*r*_(36)_ = 0.398	*r*_(36)_ = 0.24
(*n* = 39)	*p* = 0.032	*p* = 0.007	*p* = 0.073
	β = 0.04	β = 0.117	β = 0.048


*Table reflects Pearson correlation *r*, *p*, and β values from linear regression analyses of AUDIT score and AUDIT subscales score effects on ICR by recruited age groups and age groups proposed for further analysis of age effect. **BOLD:** survives Bonferroni correction for multiple comparisons (*p* < 0.0083).*

### Interacting Effect of Age and Alcohol Use in AUDIT-c Groups

**FIGURE 2:**
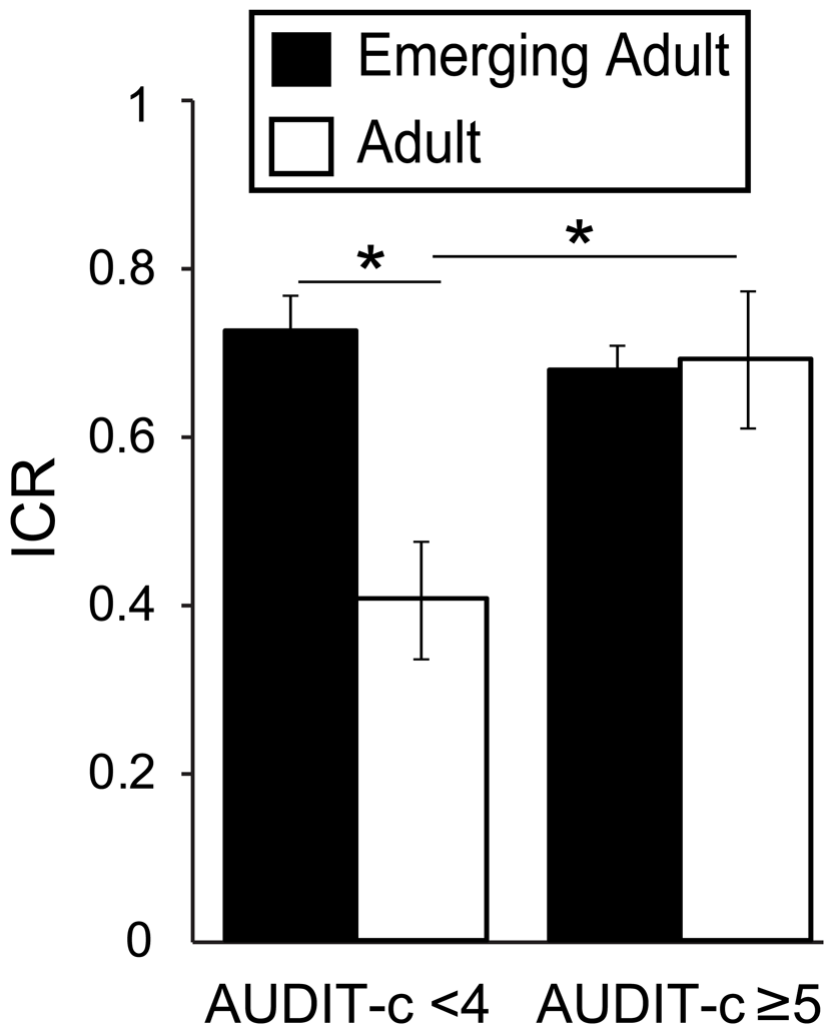
**Interacting effects of age and AUDIT-c scores on *Now* bias.** Plot depicts the ratio of immediate reward choices (ICR) in the delay-discounting task as a function of AUDIT-c (high/low) and age (adult/emerging adult) groups. There were significantly interacting effects of age and AUDIT-c on ICR, [*F*_(1,157)_ = 8.32, *p* = 0.004, η^2^ = 0.047]. This result reflects significantly higher average ICR in the high AUDIT-c versus low AUDIT-c adults [*t*_(27)_ = -2.28, *p* = 0.031, *d* = -0.88], but no difference between AUDIT-c groups among emerging adults [*t*_(115.216)_ = 1.018, *p* = 0.311, *d* = 0.17]. low AUDIT-c: <4; high AUDIT-c: ≥5; AUDIT-c, AUDIT consumption subscale. ^∗^*p* < 0.05.

Given that age related differences in *Now* bias were driven by non-heavy drinkers and that *Now* bias best correlated with AUDIT-c scores, we tested for an interaction between age group and drinking group. Taking ICR as our dependent measure, we conducted a two-way ANOVA (age group^∗^AUDIT-c) with data from emerging adults (*n* = 184; mean age = 20.8 ± 1.7) and adults (*n* = 39; mean age = 31.4 ± 4.0), classified based on AUDIT-c scores (<4, *n* = 60, mean: 2.1 ± 0.9; ≥5, *n* = 102, mean: 6.8 ± 1.6). We excluded participants with AUDIT-c scores of 4, as scores of 4 may be associated with alcohol misuse in females but not males (Bradley et al., 2007). The resulting groups were well matched, as for our initial recruitment groups (**Table 4**). As expected, we found significant main effects of age [*F*_(1,157)_ = 6.12, *p* = 0.014, η^2^ = 0.035] and AUDIT-c [*F*_(1,157)_ = 4.37, *p* = 0.038, η^2^ = 0.025] on ICR. Critically, we also observed a significant age^∗^AUDIT-c interaction on ICR [*F*_(1,157)_ = 8.32, *p* = 0.004, η^2^ = 0.047; **Figure 2**]. In the Low AUDIT-c group, mean ICR was 80% higher in emerging adults (*n* = 44, mean: 0.72 ± 0.21) relative to adults [*n* = 16, mean: 0.41 ± 0.35; *F*_(1,57)_ = 13.11, *p* = 0.001, η^2^ = 0.186]. Whereas in the High AUDIT-c group, mean ICR did not differ between age groups [*F*_(1,99)_ = 0.001, *p* = 0.98, η^2^ = 0]. Moreover, among adults, the mean ICR in the High AUDIT-c group (0.69 ± 0.32, *n* = 13) was 72.5% higher than that in the Low AUDIT-c group [0.41 ± 0.35, *n* = 16; *F*_(1,26)_ = 3.465, *p* = 0.074, η^2^ = 0.110]. In contrast, among emerging adults, mean ICR did not differ between AUDIT-c groups [*F*_(1,130)_ = 0.088, *p* = 0.721, η^2^ = 0.006]. Considering age and ICR as continuous variables, ICR negatively correlated with age in the low AUDIT-c group [*r*_(62)_ = -0.46, *p* < 0.001, β = -0.034], reflecting a 2.9% decrease in ICR with each year of age over 18. No such age effect was detected in the high AUDIT-c group [*r*_(107)_ = -0.033, *p* = 0.37, β = -0.003]. It is important to note that even if we apply much stricter statistical thresholds to these exploratory analyses, which may or may not be appropriate (Perneger, 1998), the finding of interacting effects of age and ethanol consumption levels remains significant.

**Table 4:** Demographic, substance use related, and psychometrics measures across age and high/low AUDIT-c groups.

	Light/moderate drinkers (AUDIT-c < 4)		Heavy drinkers (AUDIT-c ≥ 5)		Age^∗^AUDIT-c interaction	
			
	Ages 18–24 (*n* = 44)	Ages 26–40 (*n* = 16)	Ages 18–24 (*n* = 89)	Ages 26–40 (*n* = 13)	*F*_158_	*p*
**Demographic**						
Age (years)	20.5 ± 1.8	31.4 ± 3.8	21.1 ± 1.7	32.2 ± 4.3	0.06	0.80
Education (years)	14.5 ± 1.7	17.3 ± 1.3	14.8 ± 1.5	17.5 ± 3.4	0.002	0.97
SES	47.5 ± 11.6	52.2 ± 7.6	51.6 ± 7.5	51.7 ± 10	1.53	0.22
Ethnicity (% non-white)	20.5	31.3	30.3	38.5		0.52^†^
Sex (% female)	59.1	62.5	44.9	62.5		0.26^†^
COMT genotype (% ValVal)	20.5	18.8	28.1	46.2		0.58^†^
**Substance use-related**						
AUDIT – total	2.7 ± 2.2	2.9 ± 2.2	12.6 ± 5.2	8.7 ± 2.7	5.71	0.018
AUDIT consumption	2.1 ± 0.9	2.2 ± 0.9	7.0 ± 1.6	5.5 ± 0.7	7.62	0.006
AUDIT dependence/harm	0.8 ± 1.5	0.8 ± 1.8	5.9 ± 4.0	3.2 ± 2.5	4.23	0.041
RAPI	2.3 ± 3.2	2.1 ± 7.5	12.1 ± 7.7	6.2 ± 4.3	4.59	0.034
DUSI	0.09 ± 0.11	0.10 ± 0.14	0.39 ± 0.16	0.25 ± 0.20	6.64	0.011
DAST	0.66 ± 0.83	1.0 ± 1.2	3.0 ± 2.8	2.8 ± 4.1	0.23	0.63
FTQ density (%)	14.2 ± 16.5	25.9 ± 20.6	15.9 ± 17.3	20.3 ± 23.5	0.95	0.33
**Psychometric**						
BIS – total	56.1 ± 9.3	55.8 ± 7.6	62.0 ± 9.7	54.5 ± 9.8	3.38	0.068
BIS attention	15.3 ± 3.2	14.3 ± 3.3	16.2 ± 3.7	13.9 ± 3.5	0.69	0.41
BIS motor	20.9 ± 3.7	20.8 ± 2.1	22.7 ± 3.8	19.5 ± 3.6	4.39	0.038
BIS non-planning	19.9 ± 4.5	20.7 ± 4.4	23.1 ± 4.7	21.1 ± 4.7	2.11	0.149
FTPI mean ext (years)	8.4 ± 5.3	6.0 ± 4.5	8.0 ± 6.1	6.6 ± 5.6	0.16	0.69
FTPI max ext (years)	28.3 ± 19.8	22.2 ± 16.1	30.4 ± 24.3	22.8 ± 15.1	0.03	0.86


*Values are reported as mean ± standard deviation. Reported *F* and p-values reflect the results testing for significant age by AUDIT group interactions. Exact p-values reported unless *p* < 0.001. Conventions as per **Table 1**. ^†^*p*-value represents results of χ^*2*^ test.*

### Elevated *Now* Bias in Emerging Adults Not Driven by Underage Drinkers

The emerging adult group (ages 18–24) included participants under the U.S. legal drinking age (21 years), raising the concern that impulsive choice in the emerging adult group is driven by underage drinkers, a possibly more impulsive group. Thus, we tested for a relationship between age and ICR within non-heavy and heavy drinking groups after excluding underage participants (*n* = 71). Among non-heavy drinkers, we still observed a significant negative relationship between ICR and age [*r*_(66)_ = -0.283, *p* = 0.010; β = -0.025], reflecting a -2.4% drop in ICR with each year of age over 21. Likewise, among heavy drinkers, we still observed no relationship between age and ICR [*r*_(96)_ = -0.031, *p* = 0.38; β = -0.003]. Moreover, among non-heavy drinkers, 21–24 year olds had a mean ICR 24.9% higher than that of adults [0.67 ± 0.24 vs. 0.46 ± 0.37; *t*_(25.414)_ = 2.36, *p* = 0.026]. In addition, among 21–24 year olds, we found no significant difference in mean ICR between heavy drinkers (0.62 ± 0.32, *n* = 69) and non-heavy drinkers [0.67 ± 0.24, *n* = 44; *t*_(108.566)_ = 1.069, *p* = 0.29], and no relationship between AUDIT and ICR [*r*_(111)_ = 0.056, *p* = 0.55]. Qualitatively similar effects were found using AUDIT-c scores in correlation analyses and group definition (data not shown). Thus, high *Now* bias in our emerging adult sample is not driven by current underage drinkers.

### Age and Age × AUDIT-c Effects are Not Confounded by College Student Status

University of North Carolina students comprised 43.5% of our participants, with a significantly greater proportion of students in our emerging adult group (48.4%) than in our adult group (28.2%; χ^2^ = 5.29, *df* = 1, *p* = 0.021). Therefore, we repeated our key analyses above covarying for student status, which did not alter our age^∗^AUDIT-c findings. We detected significant main effects on ICR of age group [*F*_(1,156)_ = 5.94, *p* = 0.016, η^2^ = 0.034], AUDIT-c group [*F*_(1,156)_ = 4.34, *p* = 0.039, η^2^ = 0.025], and a significant age^∗^AUDIT-c interaction [*F*_(1,156)_ = 8.14, *p* = 0.005, η^2^ = 0.047]. Thus differences in the proportion of students between groups cannot account for the age effects on *Now* bias observed here.

### Family History of Alcoholism and *Now* Bias

**FIGURE 3:**
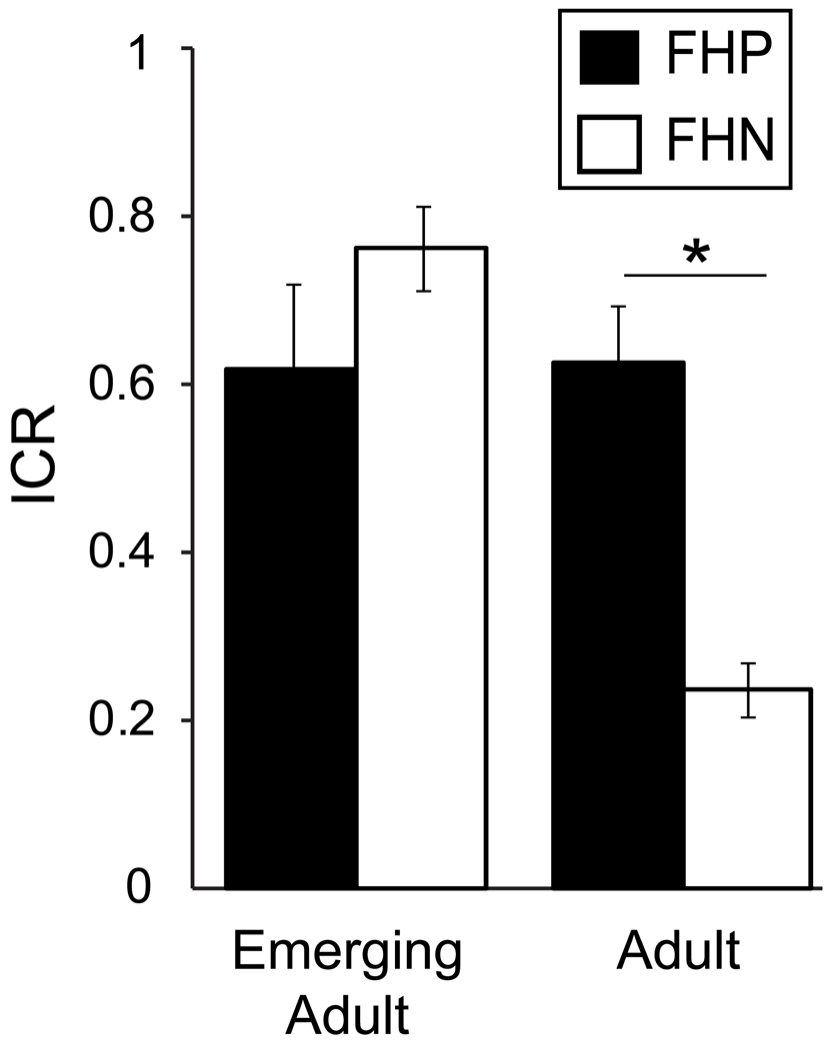
**Family history (FH) of an alcohol use disorder (AUD) is associated with greater *Now* bias in non-heavy drinking adults but not emerging adults.** Age group and FH had an interacting effect on ICR [*F*_(1,50)_ = 16.40, *p* < 0.001, η^2^ = 0.203]. This interaction reflects dramatically higher ICRs among adults with a first degree relative with an AUD (FHP) relative to adults with no first degree relative with an AUD [FHN; *F*_(1,12)_ = 7.21, *p* = 0.020, η^2^ = 0.366] and a smaller, opposing trend effect of FH among emerging adults [*F*_(1,37)_ = 3.89, *p* = 0.056, η^2^ = 0.092]. ^∗^*p* < 0.05.

Our findings thus far do not point to heightened *Now* bias as a pre-existing trait associated with AUD risk, as it was present generally among emerging adults regardless of drinking behavior. However, another way to identify intermediate phenotypes for complex neurobehavioral disorders is to compare the behavior of unaffected people with and without 1° relatives with the disorder (Meyer-Lindenberg and Weinberger, 2006). As our sample included a significant proportion of family history positive (FHP) participants (∼23%), we evaluated the effect of FH (see Materials and Methods) on ICR among non-heavy drinkers stratified by age group. We found a trend toward a main effect of FH with higher ICRs in FHP (0.62 ± 0.25) relative to FHN (0.50 ± 0.31) participants [*F*_(1,50)_ = 3.52, *p* = 0.066, η^2^ = 0.044], but we also found a significant age^∗^FH interaction [*F*_(1,50)_ = 16.4, *p* < 0.001, η^2^ = 0.203; **Figure 3**]. This interaction reflects dramatically higher ICRs among FHP adults (0.61 ± 0.27) relative to FHN adults [0.23 ± 0.32; *F*_(1,12)_ = 7.21, *p* = 0.020, η^2^ = 0.366] and smaller, opposing effects of FH among emerging adults, as reported above. In fact, ICR values of non-heavy drinking FHP adults are quantitatively similar to those reported here for heavy drinking adults (**Figures 1**, and **2**) and in adults with AUDs (Mitchell et al., 2005; Boettiger et al., 2007).

### Participants Possibly Meeting DSM Criteria for Alcohol Dependence

While none of our participants self-identified as a problem drinkers, we conducted a *post hoc* evaluation of responses to each item on the RAPI, which suggested that 43 recruited subjects may have met *DSM–IV* criteria for alcohol dependence (American Psychiatric Association, 1994). To evaluate the effect of these individuals on our findings, we removed them and repeated our key analyses. When excluding these individuals, we still observed a main effect of age group [*F*_(1,177)_ = 5.98, *p* = 0.015, η^2^ = 0.031] and a significant age*heavy drinker interaction [*F*_(1,177)_ = 5.27, *p* = 0.023, η^2^ = 0.028]. Our *post hoc* age^∗^AUDIT-c findings also stood, with significant main effects of age [*F*_(1,126)_ = 6.57, *p* = 0.012, η^2^ = 0.045] and AUDIT-c [*F*_(1,126)_ = 4.80, *p* = 0.03, η^2^ = 0.033], and a significant age*AUDIT-c interaction [*F*_(1,126)_ = 7.79, *p* = 0.006, η^2^ = 0.054]. Our FH findings were also robust to excluding these individuals. Within the low AUDIT-c group, we still observed a trend toward a FH effect on ICR [*F_(_*_1,48)_ = 3.49, *p* = 0.068, η^2^ = 0.046] and a significant age^∗^FH interaction on ICR [*F*_(1,48)_ = 15.279, *p* < 0.001, η^2^ = 0.199]. Moreover, among the adult low AUDIT-c group, we observed significantly higher ICRs among FHP individuals (0.62 ± 0.29) than among FHN individuals [0.23 ± 0.32; *F*_(1,11)_ = 7.12, *p* = 0.022, η^2^ = 0.378]. Surprisingly, we observed an opposing effect of FH among emerging adults, finding significantly higher ICRs in FHN individuals (0.76 ± 0.19) than in FHP individuals [0.62 ± 0.25, *F_(_*_1,36)_ = 4.35, *p* = 0.044, η^2^ = 0.105].

## Discussion

### *Now* Bias Across the Lifespan

Taking together our findings (Mitchell et al., 2005; Kelm et al., 2010) and those from other laboratories (Green et al., 1994; Scheres et al., 2006; Olson et al., 2007; Eppinger et al., 2012), we hypothesized reduced *Now* bias as a function of age in non-heavy drinkers within the emerging adult to adult age range (18–40). Here we demonstrate that *Now* bias indeed negatively correlates with age among non-heavy drinkers, an effect driven by FHN individuals. We note that a prior study also found a significant negative correlation between *Now* bias and age among non-problem drinkers, but not among alcoholics (Dom et al., 2006); however, the mean age of non-problem drinkers in that sample was 41 years, and no information was given regarding participant age range. These data, together with our present findings suggest *Now* bias normally declines with age, but that heavy, even sub-clinical, drinking may uncouple this relationship. Longitudinal studies are needed to directly test this hypothesis. The heightened *Now* bias we observed among emerging adults, indicates that *Now* bias is only useful as an intermediate phenotype in adults.

### *Now* Bias and Alcohol Use

Elevated *Now* bias is consistently observed among people with AUDs versus those without (Petry, 2001; Bjork et al., 2004; Mitchell et al., 2005, 2007; Boettiger et al., 2007). Here, in a non-clinical sample, we also observed greater *Now* bias among heavy drinking adults, but not emerging adults. These results would appear to conflict with those of (Vuchinich and Simpson, 1998), which measured *Now* bias in emerging adults, finding greater *Now* bias among problem drinkers than social drinkers. This contradiction could reflect study differences in both drinking and *Now* bias measures. That study was also rather small (*n* = 31), and nearly all problem drinkers were under age 20, while the social drinker’s average age was over 20. Thus, age effects on *Now* bias may have led to apparent drinking status effects.

In a mixed population of people with or without AUDs we previously found ICR to positively correlate with AUDIT dependence and harm subscales, but not AUDIT-c (Mitchell et al., 2005). In contrast, in this sample with less variance in the AUDIT dependence and harm scales, AUDIT-c scores best predicted ICR. In adults, AUDIT-c scores of 5 or higher were associated with elevated *Now* bias on a par with that of adults with AUDs (Mitchell et al., 2005; Boettiger et al., 2007), suggesting that heightened *Now* bias in adults may be an early AUD risk indicator. Among emerging adults, however, putative developmental effects on choice behavior appear to occlude any relationship between alcohol use and *Now* bias, except in FHP individuals, in whom AUDIT-c scores also positively correlated with *Now* bias.

### Impulsivity in Emerging Adults and Heavy Drinking Adults: Shared Mechanisms?

Our data demonstrate equivalent *Now* bias in two groups at risk for developing AUDs: emerging adults and heavy drinking adults. The lack of additive effects of heavy drinking and age suggests the possibility of shared underlying mechanisms in these populations. For example, both groups might have dysfunction in the frontal circuitry engaged during *Now/Later* decision-making (Boettiger et al., 2007). In support of this idea, frontal development is incomplete until the early-to-mid twenties (Sowell et al., 1999, 2001; Casey et al., 2000; Giedd, 2004; Gogtay et al., 2004; Lenroot and Giedd, 2006), and functional brain maturation continues into the mid-to-late 20s (Dosenbach et al., 2010). Such maturational effects are consistent with our finding that “adult-like” choice behavior is present by the mid-20s. Moreover, a structural neuroimaging study of individuals aged 9–23, found age-related increases in frontal white matter integrity to negatively correlate with *Now* bias (Olson et al., 2009); however, that study included very few individuals over 18, precluding conclusions regarding changes from emerging adulthood to adulthood. Recent functional neuroimaging data show that changes in corticostriatal circuit function from early adolescence (age 11) to early adulthood (age 31) are associated with decreases in *Now* bias (Christakou et al., 2011). Thus, changes in frontal circuits could underlie the negative relationship between age and *Now* bias that we observed among FHN non-heavy drinkers. Not only do frontal circuits mature late, they are especially prone to alcohol insult, especially binge drinking (De Bellis et al., 2005; Miguel-Hidalgo et al., 2006; Jacobus et al., 2009; McQueeny et al., 2009; Vargas et al., 2014), and decreased frontal metabolism is observed in AUDs (Volkow et al., 1997; Catafau et al., 1999). Thus, *Now* bias could reflect immature frontal function in emerging adults, and dysfunction in similar circuits in heavy drinking adults.

### *Now* Bias Indices and Delay Discounting Task Designs

A great variety of intertemporal choice tasks are in current use. The task employed here (Mitchell et al., 2005) has several advantages over adjusting amount procedures, which are used to calculate individual indifference points (Madden et al., 1997; Richards et al., 1999). First, our task includes objective choice conditions that allow us to detect patterns of invalid responding. We can detect inaccurate performance in these control conditions, and we can also compare RTs across trial types to determine whether participants are making a subjective decision during our “WANT” condition, which should take longer than simple objective choices in our control trials. Participants not meeting these criteria can be removed from our analyses, ensuring that all choice data are from participants who adhered to task instructions. Also, our design randomly varies the delayed amount, delay time, discount between choices, and trial type, which reduces interference from previous choices. This differs from adjusting amount procedures where options are titrated based on participants’ prior choice (e.g., by incrementally increasing and decreasing the immediate reward amount), which may bias choices. This task is also fMRI-compatible (Boettiger et al., 2007, 2009), allowing for investigation into the neural correlates of our behavioral findings.

### *Now* Bias as an AUD Intermediate Phenotype

Determining the biological bases of complex neurobehavioral disorders like AUDs is difficult, whereas the biological etiology should be clearer for simpler, intermediate phenotypes (Meyer-Lindenberg and Weinberger, 2006). As has been recently argued (MacKillop, 2013), *Now* bias in adults shows promise as an intermediate phenotype for AUDs. First, *Now* bias bears an obvious relationship to AUDs, as every relapse or excess drink constitutes a decision favoring immediate over delayed benefits. Second, *Now* bias behavior has good psychometric properties: ICR is a highly reliable measure (Chronbach’s *α* > 0.98). Third, as we show here, *Now* bias is also elevated in unaffected first degree relatives. Fourth, *Now* bias appears to be heritable (Anokhin et al., 2011). Finally, *Now* bias appears to be stable over time (Kirby, 2009), although our data suggest that slow timescale changes may occur particularly between adolescence and adulthood. Evidence regarding cosegregation of *Now* bias with AUDs within families is currently lacking, but every other criteria for an intermediate phenotype appears to be met (Meyer-Lindenberg and Weinberger, 2006; MacKillop, 2013).

Prior studies investigating the relationship between FH and *Now* bias have either found little to no relationship in small samples (Crean et al., 2002; Herting et al., 2010), or greater delayed reward discounting in FHP individuals in larger sized samples (Acheson et al., 2011; Dougherty et al., 2014). It is possible that the increased power needed to detect a FH effect on *Now* bias in the prior studies is the concentration of adolescents or young adults in prior samples. If youth is associated with heightened *Now* bias as we and others (Green et al., 1994; Scheres et al., 2006; Olson et al., 2007; Eppinger et al., 2012) have found, age effects may hinder the detection of FH effects in all but very large samples. Indeed, a study with roughly half the participants of more recent ones but with an average age around 26 found effects of FH on *Now* bias in females (Petry et al., 2002). That Petry et al. (2002) observed FH effects only in females supports the idea that developmental “maturity” may be needed to see FH effects in moderate sample sizes, as female brains reach functional maturity a bit earlier than those of males (Lenroot et al., 2007; Lenroot and Giedd, 2010). Thus, the brains of Petry et al.’s (2002) female participants may have been sufficiently mature to allow detection of FH effects, while the brains of the males (in their early-to-mid 20s) may not have been. An important take home point here is the importance of considering how age effects may occlude detection of the effects of other factors on *Now* bias. Future studies directly testing the effect of FH on *Now* bias in adults are needed to confirm that *Now* bias meets this intermediate phenotype criterion.

Finally, all FH studies mentioned here that found some FH effect on delay discounting behavior (Petry et al., 2002; Acheson et al., 2011; Dougherty et al., 2014) used the 27-item Monetary Choice Questionnaire (Kirby and Marakovic, 1996; Kirby et al., 1999), while those employing a behavioral choice task (Crean et al., 2002; Herting et al., 2010) did not detect FH effects. Our results suggest that in behavioral choice tasks, age and FH interact to affect *Now* bias, which may have prevented Crean et al. (2002) and Herting et al. (2010) from detecting robust FH effects. Further work investigating whether age and FH modulate questionnaire-based measures in a similar manner might highlight potential differences in the choice processes tapped in differing delay discounting assessment procedures.

### Study Limitations

While our recruitment group age ranges were evidence-based and yielded a main effect of age group on *Now* bias in non-heavy drinkers, our data show elevated *Now* bias up to about age 25. Our smaller sample size in the adult group limited our power to investigate effects of other factors on *Now* bias (e.g., sex) in adults. Second, although we excluded individuals with self reported AUDs, we did not directly test for AUD diagnoses, and some people with AUDs may have been included. Third, AUDIT and AUDIT-c scores are a coarse measure of alcohol use. More detailed measures of drinking behavior may have established how the quantity, frequency, or pattern of alcohol use may associate with *Now* bias. For example, binge drinking may better predict *Now* bias, as it is most damaging to the brain and frontal cortices in particular. Finally, while our results suggest that *Now* bias decreases with age in non-heavy drinkers, this study was cross-sectional, which precludes drawing any developmental conclusions. Longitudinal studies are needed to directly confirm this idea.

## Conclusion

Here, we found that three factors associated with increased AUD risk also associate with elevated *Now* bias: age, heavy alcohol use, and FH status. *Now* bias negatively correlates with age in FHN non-heavy drinkers, declining to adult levels around the mid-twenties. In adults, we found *Now* bias equivalent to that seen in abstinent alcoholics in both heavy drinkers and in FHP individuals. These data support the idea that elevated *Now* bias may be an intermediate phenotype for AUDs that might serve as an early warning sign *in adults*. The underlying neural bases of elevated *Now* bias in emerging adults, heavy drinkers, and FHP individuals remain to be identified.

## Conflict of Interest Statement

The Reviewer Bradley Conner declares that, despite being affiliated with the same institution as the Associate Editor Carol Seger, the review process was handled objectively. The authors declare that the research was conducted in the absence of any commercial or financial relationships that could be construed as a potential conflict of interest.

## Supplementary Material

FIGURE S1 **Systematic analysis of adult age group cutoff on ICR age group effects among non-heavy drinkers.** Plot depicts the group discrimination index (d′) as a function of adult age cutoff. Among non-heavy drinkers in this sample, d′ was maximal with an adult age cutoff of 26 years of age. Note that a gap year was included in each age group comparison.
