# Supplementary material for: Intertemporal Choice Behavior in Emerging Adults and Adults: Effects of Age Interact with Alcohol Use and Family History Status
Source: Front Hum Neurosci. 2015 Nov 23;9:627. doi: 10.3389/fnhum.2015.00627 (PMC4655234; doi:10.3389/fnhum.2015.00627)
Supplement: Supplementary file 2 [file Table_1.DOCX]

Supplementary Material

**Intertemporal choice behavior in emerging adults and adults: effects of age interact with alcohol use and family history status**

**Christopher T. Smith, Eleanor A. Steel, Michael H. Parrish, Mary Katherine Kelm, Charlotte A. Boettiger^*^**

*** Correspondence:** Charlotte Boettiger: cab@unc.edu

**Supplementary Table 1. Demographic, substance use, and psychometric data by age recruitment group.**

|  | Ages 18-21  (*n* = 111) | Ages 22-40  (*n* = 126) | *t* _(235)_ | *p value* |
| --- | --- | --- | --- | --- |
| *General* |  |  |  |  |
| Age (yrs) | 19.7 ± 1.2 | 25.5 ± 4.6 | -13.58 | <0.001 |
| Education (yrs) | 13.8 ± 1.2 | 16.5 ± 1.7 | -13.17^a^ | <0.001 |
| SES | 51.4 ± 8.2 | 51.1 ± 8.9 | 0.20 | 0.84 |
| Gender (% female) | 49.5 | 50.8 |  | 0.85^†^ |
| Ethnicity (% non-white) | 33.3 | 24.6 |  | 0.14^†^ |
| COMT genotype (% ValVal) | 22.5 | 31.7 |  | 0.32^†^ |
|  |  |  |  |  |
| *Substance use-related* |  |  |  |  |
| AUDIT - total | 9.2 ± 6.5 | 7.4± 4.6 | 2.41 | 0.017 |
| AUDIT consumption | 4.9 ± 2.6 | 4.8 ± 2.2 | 0.44^b^ | 0.66 |
| AUDIT dependence/harm | 4.2 ± 4.4 | 3.0 ± 3.1 | 2.34^b^ | 0.02 |
| RAPI | 8.8 ± 8.0 | 6.7 ± 7.4 | 2.16 | 0.032 |
| DUSI | 0.3 ± 0.2 | 0.3 ± 0.2 | 1.60 | 0.11 |
| DAST | 2.1 ± 2.5 | 2.0 ± 2.4 | 0.43 | 0.67 |
| FTQ density (%) | 16.9 ± 18.0 | 14.8 ± 16.6 | 0.93 | 0.356 |
|  |  |  |  |  |
| *Psychometric* |  |  |  |  |
| BIS - total | 60.5 ± 9.6 | 57.8 ± 9.6 | 2.17^c^ | 0.031 |
| BIS Attention | 15.9 ± 3.7 | 15.3 ± 3.5 | 1.3^c^ | 0.19 |
| BIS Motor | 22.1 ± 3.5 | 21.3 ± 3.8 | 1.74^c^ | 0.084 |
| BIS Non-Planning | 22.4 ± 4.6 | 21.2 ± 4.6 | 2.12^c^ | 0.035 |
| FTPI mean extension (yrs) | 8.6 ± 5.4 | 6.6 ± 5.4 | 2.89 | 0.004 |
| FTPI max extension (yrs) | 31.1 ± 23.3 | 24.4 ± 19.9 | 2.37 | 0.018 |

Values are reported as mean ± standard deviation. Reported *p*-values reflect the results of unpaired two-tailed comparison between groups. Exact *p*-values reported unless *p* < 0.001. AUDIT, Alcohol Use Disorders Identification Test; RAPI, Rutgers Alcohol Problem Index; DUSI, Drug Use Screening Inventory, part I-B; DAST, Drug Abuse Screening Test; SES, Socioeconomic Status; BIS, Barratt Impulsiveness Scale; FTPI, Future Time Perspective Inventory. ^†^*p*-value represents results of *χ^2^* test. ^a^*df*=233, ^b^*df*=215; ^c^*df*=234
