# Supplementary material for: Intertemporal Choice Behavior in Emerging Adults and Adults: Effects of Age Interact with Alcohol Use and Family History Status
Source: Front Hum Neurosci. 2015 Nov 23;9:627. doi: 10.3389/fnhum.2015.00627 (PMC4655234; doi:10.3389/fnhum.2015.00627)
Supplement: Supplementary file 3 [file Table_2.DOCX]

Supplementary Material

**Intertemporal choice behavior in emerging adults and adults: effects of age interact with alcohol use and family history status**

**Christopher T. Smith, Eleanor A. Steel, Michael H. Parrish, Mary Katherine Kelm, Charlotte A. Boettiger^*^**

*** Correspondence:** Charlotte Boettiger: cab@unc.edu

**Supplementary Table 2. Demographic, substance use, and psychometric data by drinking recruitment group**

|  | Non-heavy Drinkers  (AUDIT <8 M, <5 F)  *n*=99 | Heavy Drinkers  (AUDIT ≥8 M, ≥5 F)  *n*=138 | *t* _(235)_ | *p value* |
| --- | --- | --- | --- | --- |
| *General* |  |  |  |  |
| Age (yrs) | 23.0 ± 4.8 | 22.7 ± 4.3 | 0.55 | 0.58 |
| Education (yrs) | 15.4 ± 2.0 | 15.2 ± 2.0 | 0.76^a^ | 0.45 |
| SES | 51.1 ± 9.4 | 51.4 ± 8.0 | -0.27 | 0.79 |
| Gender (% female) | 44.4 | 54.3 |  | 0.13^†^ |
| Ethnicity (% non-white) | 29.3 | 28.3 |  | 0.86^†^ |
| COMT genotype (% ValVal) | 24.2 | 29.7 |  | 0.63^†^ |
|  |  |  |  |  |
| *Substance use-related* |  |  |  |  |
| AUDIT - total | 3.4 ± 2.1 | 11.8 ± 4.7 | -18.77 | <0.001 |
| AUDIT consumption | 2.9 ± 1.5 | 6.3 ± 1.9 | -13.4^a^ | <0.001 |
| AUDIT dependence/harm | 0.8 ± 1.2 | 5.7 ± 3.7 | -14.0^a^ | <0.001 |
| RAPI | 2.6 ± 4.0 | 11.3 ± 7.7 | -11.5 | <0.001 |
| DUSI | 0.1 ± 0.1 | 0.4 ± 0.2 | -13.72 | <0.001 |
| DAST | 1.0 ± 1.3 | 2.8 ± 2.8 | -6.7 | <0.001 |
| FTQ density (%) | 14.9 ± 16.5 | 16.5 ± 17.8 | -0.72 | 0.475 |
|  |  |  |  |  |
| *Psychometric* |  |  |  |  |
| BIS - total | 56.7 ± 8.9 | 60.8 ± 9.9 | -3.23^b^ | 0.001 |
| BIS Attention | 15.2 ± 3.3 | 15.9 ± 3.9 | -1.55^b^ | 0.122 |
| BIS Motor | 21.2 ± 3.4 | 22.1 ± 3.9 | -1.88^b^ | 0.062 |
| BIS Non-Planning | 20.4 ± 4.4 | 22.8 ± 4.5 | -4.05^b^ | <0.001 |
| FTPI mean extension (yrs) | 7.8 ± 5.3 | 7.3 ± 5.6 | 0.62 | 0.54 |
| FTPI max extension (yrs) | 27.9 ± 20.9 | 27.3 ± 22.4 | 0.21 | 0.84 |

Values are reported as mean ± standard deviation. Reported *p*-values reflect the results of unpaired two-tailed comparison between groups. Exact *p*-values reported unless *p* < 0.001. Conventions as per Supplementary Table 1. ^†^*p*-value represents results of *χ^2^* test. ^a^*df*=233, ^b^*df*=215; ^c^*df*=234
