# Supplementary material for: Intertemporal Choice Behavior in Emerging Adults and Adults: Effects of Age Interact with Alcohol Use and Family History Status
Source: Front Hum Neurosci. 2015 Nov 23;9:627. doi: 10.3389/fnhum.2015.00627 (PMC4655234; doi:10.3389/fnhum.2015.00627)
Supplement: Supplementary file 4 [file Table_3.DOCX]

Supplementary Material

**Intertemporal choice behavior in emerging adults and adults: effects of age interact with alcohol use and family history status**

**Christopher T. Smith, Eleanor A. Steel, Michael H. Parrish, Mary Katherine Kelm, Charlotte A. Boettiger^*^**

*** Correspondence:** Charlotte Boettiger: cab@unc.edu

**Supplementary Table 3. Demographic, Substance Use Related, and Psychometrics Measures Across Age and Drinking recruitment groups**

|  | Non-heavy drinkers | | Heavy drinkers | |  |  |
| --- | --- | --- | --- | --- | --- | --- |
|  | Ages 18-21  (*n* = 45) | Ages 22-40  (*n* = 54) | Ages 18-21  (*n* = 66) | Ages 22-40  (*n* = 72) | Age*drinking interaction | |
|  |  |  |  |  | *F*_233_ | *p* |
| *Demographic* |  |  |  |  |  |  |
| Age (yrs) | 19.6 ± 1.2 | 25.8 ± 4.9 | 19.8 ± 1.1 | 25.3 ± 4.4 | 0.85 | 0.36 |
| Education (yrs) | 13.8 ± 1.4 | 16.7 ± 1.4 | 13.9 ± 1.2 | 16.3 ± 2.0 | 1.08^a^ | 0.30 |
| SES | 51.6 ± 9.2 | 50.6 ± 9.6 | 51.2 ± 7.6 | 51.5 ± 8.4 | 0.31 | 0.58 |
| Ethnicity (% non-white) | 31.1 | 27.8 | 34.8 | 22.2 |  | 0.42^†^ |
| Sex (% female) | 42.2 | 46.3 | 54.5 | 54.2 |  | 0.49^†^ |
| COMT genotype  (% ValVal) | 20 | 27.8 | 24.2 | 34.7 |  | 0.59^†^ |
|  |  |  |  |  |  |  |
| *Substance use-related* |  |  |  |  |  |  |
| AUDIT | 3.3 ± 2.3 | 3.4 ± 1.9 | 13.2 ± 5.2 | 10.5 ± 3.6 | 7.91 | 0.005 |
| AUDIT consumption | 2.7 ± 1.4 | 3.1 ± 1.5 | 6.5 ± 2.0 | 6.0 ± 1.8 | 2.98^b^ | 0.086 |
| AUDIT dependence/harm | 0.8 ± 1.1 | 0.7 ± 1.2 | 6.8 ± 4.2 | 4.8 ± 2.9 | 5.92^b^ | 0.016 |
| RAPI | 3.3 ± 4.7 | 1.9 ± 3.1 | 12.6 ± 7.5 | 10.2 ± 7.7 | 0.38 | 0.54 |
| DUSI | 0.12 ± 0.13 | 0.13 ± 0.12 | 0.41 ± 0.15 | 0.34 ± 0.16 | 3.97 | 0.048 |
| DAST | 1.02 ± 1.5 | 0.91 ± 1.1 | 2.8 ± 2.9 | 2.7 ± 2.8 | 0.003 | 0.96 |
| FTQ density (%) | 15.1 ± 16.6 | 14.7 ± 16.6 | 18.2 ± 18.9 | 15 ± 16.7 | 0.359 | 0.55 |
|  |  |  |  |  |  |  |
| *Psychometric* |  |  |  |  |  |  |
| BIS - total | 57.6 ± 8.8 | 56 ± 9.1 | 62.5 ± 9.7 | 59.2 ± 9.9 | 0.44^c^ | 0.51 |
| BIS Attention | 15.3 ± 3.3 | 15.1 ± 3.3 | 16.4 ± 3.9 | 15.5 ± 3.7 | 0.69^c^ | 0.41 |
| BIS Motor | 21.7 ± 3.3 | 20.7 ± 3.4 | 22.4 ± 3.7 | 21.8 ± 4.0 | 0.11^c^ | 0.74 |
| BIS Non-Planning | 20.7 ± 4.1 | 20.1 ± 4.7 | 23.7 ± 4.5 | 22 ± 4.4 | 1.004^c^ | 0.32 |
| FTPI mean ext (yrs) | 9.0 ± 5.0 | 6.7 ± 5.4 | 8.3 ± 5.8 | 6.4 ± 5.3 | 0.134 | 0.72 |
| FTPI max ext (yrs) | 32.3 ± 22.1 | 24.2 ± 19.3 | 30.2 ± 24.3 | 24.6 ± 20.4 | 0.188 | 0.67 |

Values are reported as mean ± standard deviation. Reported *p*-values reflect the results of unpaired two-tailed comparison between groups. Exact *p*-values reported unless *p* < 0.001. Conventions as per Supplementary Table 1. ^†^*p*-value represents results of *χ^2^* test. ^a^*df*=231; ^b^*df*=213, ^c^*df*=232
